# Morphological and phylogenetic evidence for two new species of *Russula* subg. *Heterophyllidia* from Guangdong Province of China

**DOI:** 10.3897/mycokeys.82.64913

**Published:** 2021-08-11

**Authors:** Bin Chen, Jie Song, Yanliu Chen, Jinhua Zhang, Junfeng Liang

**Affiliations:** 1 Research Institute of Tropical Forestry, Chinese Academy of Forestry, Guangzhou 510520, China Research Institute of Tropical Forestry, Chinese Academy of Forestry Guangzhou China; 2 Nanjing Forestry University, Nanjing 210037, China Nanjing Forestry University Nanjing China

**Keywords:** Luofu Mountain, new species, phylogeny, Russulaceae, taxonomy

## Abstract

Two new species of Russulasubg.Heterophyllidia from Guangdong Province of China were described and illustrated based on morphological characters, and their identity supported by molecular phylogeny. *R.luofuensis* is morphologically characterized by a grayish yellow to brownish orange pileus center with a purplish gray to grayish magenta margin, a surface that is cracked and broken into small golden-brown patches, subglobose to broadly ellipsoid basidiospores with warts fused in short or long chains and a suprapellis composed of hyphal extremities with inflated, ellipsoid or globose cells and attenuated terminal cell. *R.subbubalina* is distinguished by the blanched almond to dark salmon pileus that is cracked with age, subglobose to broadly ellipsoid basidiospores with wart fused in short or long chains and frequently connected by line connections, a suprapellis with hyphal ends composed of inflated or ellipsoid cells and attenuated terminal cell, and pileocystidia that are mainly clavate and sometimes with round or ellipsoid appendage. The phylogenetic analyses based on ITS-nrLSU-mtSSU-*TEF1* dataset were performed using maximum likelihood and Bayesian analysis. In terms of morphological features and molecular data, the former species belongs to subsect. Virescentinae, whereas the latter comes under subsect. Heterophyllinae.

## Introduction

*Russula* Pers. is the largest genus of Russulaceae, estimated at least to contain 2000 species, which has resulted in many complex and multilevel classifications (Buyck et al. 2018; Adamčík et al. 2019; Wijayawardene et al. 2020). Recent molecular phylogenetic studies have indicated eight subgenera within the genus R.subg.Glutinosae Buyck & X.H. Wang, R.subg.Archaeae Buyck & V. Hofst., R.subg.Compactae (Fr.) Bon, R.subg.Crassotunicatae Buyck & V. Hofst., R.subg.Heterophyllidiae Romagnesi, R.subg.Malodorae Buyck & V. Hofst., R.subg.Brevipedum Buyck & V. Hofst., and R.subg.Russula (Buyck et al. 2018, 2020). The infrageneric classification system of *Russula* based on a multi-locus phylogenetic analysis was followed in this study. This genus is globally distributed and occurs across a wide range of habitats from Arctic tundra to tropical forests and forms ectomycorrhizal relationships with diverse plants (Knudsen and Borgen 1982; Buyck et al. 1996; Looney et al. 2018). Some species of *Russula* are famous edible fungi and are also commercially traded worldwide (Looney et al. 2018; Wang 2020). According to recent statistics on the resource diversity of Chinese macrofungi, there are 78 edible *Russula* species in China (Wu et al. 2019).

Guangdong Province is located in the southern coastal area of China, which is one of the Chinese provinces with tropical and subtropical climates. The climate can be divided into the middle subtropical, the southern subtropical, and the tropical climate zones, from north to south. The annual average temperature in Guangdong Province is 19–24 °C and the annual average rainfall is 1500–2000 mm. Abundant moisture, moderate to high temperatures, and variegated physiography support luxuriant and highly diversified plant growth. Broad-leaved evergreen forests, intermixed with coniferous and deciduous trees, cover much of the land. During the rainy season, the forest ecosystem can facilitate the fruiting of most ectomycorrhizal fungi, among which the members of *Russula* are very common. Recently, 16 new species and one epitype of *Russula* from Guangdong Province have been reported (Das et al. 2017; Zhang et al. 2017; Song et al. 2018a, b; Li et al. 2019; Yuan et al. 2019; Zhou et al. 2020). Obviously, Guangdong Province has become a hotspot in research on biodiversity of Chinese *Russula*, which makes it more vital for us to continue to explore it.

Northern hemisphere species within subg. Heterophyllidia are mainly characterized by the mostly medium to large basidiomata, equal lamellae, mild to strongly acrid taste, white or cream and rarely ochre spore print, basidiospores with inamyloid or partly amyloid suprahilar spot, mostly abundant gloeocystidia that are typically mucronate to obtuse-rounded, and absence of primordial hyphae. During a survey of the habitat diversity and geographic distribution of *Russula* in Guangdong Province, some interesting specimens of subg. Heterophyllidia were found that were different from known species. In this study, two new species from Guangdong Province are presented based on the morphological characters and molecular data.

## Materials and methods

### Morphological study

Fresh specimens were collected and photographed in Luofu Mountain Provincial Nature Reserve, Guangdong Province, South China. Collections were dried at 45–55 °C and deposited in the herbarium of the Research Institute of Tropical Forestry, Chinese Academy of Forestry (RITF). The macromorphological characters were described based on detailed notes and photographs. The color codes mostly refer to Kornerup and Wanscher (1981). The description templates and terminology of the micromorphological characters were taken from Adamčík et al. (2019). Estimates of spore ornamentation density from scanning electron microscopy pictures follow Adamčík and Marhold (2000). The hymenial cystidia density estimates refer to Buyck (1991). Experiments were performed on dried specimens using a ZEISS Imager M2 (Carl Zeiss AG; Germany). The basidiospores were observed and measured in Melzer’s reagent from a lateral view excluding ornamentation. After pretreatment of dried specimens in 5% potassium hydroxide (KOH), other micromorphological characters were identified and measured in Congo red. The coloring of the cystidia contents was observed in a sulfovanillin (SV) solution (Caboň et al. 2017). The pileipellis were examined in cresyl blue to verify the presence of ortho- or metachromatic reactions (Buyck 1989). The structure and ornamentation of the basidiospores were illustrated using a scanning electron microscopy (SEM-JEOL JSM-6510). Basidiospore measurements are presented as (Min–)AV-SD–AV–AV+SD(–Max), where Min is the minimum value, Max is the maximum value, AV is the average value, SD is the standard deviation, and Q represents the length/width ratio of the basidiospores.

### Molecular study

The total genomic DNA was extracted from dry specimens following an improved CTAB protocol (Zhou and Liang 2011). We amplified and sequenced the following four loci with standard primer sets: 600 base pairs of the ITS region of rDNA using the primers ITS1 and ITS4 (White et al. 1990); 900 base pairs of the nuclear ribosome large subunit (nrLSU) using the primers LROR and LR5 (Vilgalys and Hester 1990); 600 base pairs of the ribosomal mitochondrial small subunit (mtSSU) with primers MS1 and MS2 (White et al. 1990); 900 base pairs of the translation elongation factor 1-alpha (TEF1) using primers EF1-F and EF1-R (Morehouse et al. 2003). Successful PCR products were subjected to automated DNA sequencing on an ABI 3730 DNA analyser using an ABI BigDye 3.1 terminator cycle sequencing kit (Shanghai Sangon Biological Engineering Technology and Services CO., Ltd, Shanghai, China). The newly generated sequences were submitted to GenBank database (Table 1).

**Table 1.** GenBank accession numbers for sequences used in phylogenetic tree. The newly generated sequences are in bold.

Taxon	Voucher	Location	ITS	nrLSU	mtSSU	*TEF1*	Reference
* R. aeruginea *	AT2003017	Sweden	DQ421999	DQ421999	‒	‒	Buyck et al. 2008
* R. albidogrisea *	K15091234	China	KY767807	‒	‒	MN617847	Das et al. 2017
* R. albidogrisea *	RITF1871	China	MW397095	MW397128	MW403841	‒	Unpublished
* R. amoena *	SAV F–3147	Slovakia	MT017544	‒	MT417190	MT417211	Wisitrassameewong et al. 2020
* R. aureoviridis *	H16082612	China	KY767809	‒	‒	MN617846	Das et al. 2017
* R. aureoviridis *	RITF4709	China	**MW646980**	**MW646992**	**MW647003**	**MW650849**	This work
* R. bella *	SFC20170819-05	South Korea	MT017552	‒	MT196931	MT199655	Wisitrassameewong et al. 2020
* R. bubalina *	K15052614	China	MG018742	‒	‒	‒	Li et al. 2019
* R. bubalina *	RITF1863	China	MW397097	‒	MW403843	‒	Unpublished
* R. crustosa *	BPL265	USA	KT933966	KT933826	‒	‒	Looney et al. 2016
* R. cyanoxantha *	FH 12-201	Germany	KR364093	KR364225	‒	‒	De Crop et al. 2017
* R. cyanoxantha *	RITF4682	China	**MW646981**	**MW646993**	**MW647004**	‒	This work
* R. dinghuensis *	GDGM45244	China	KU863579	‒	‒	MN617848	Zhang et al. 2017
* R. dinghuensis *	RITF5142	China	**MW646982**	**MW646994**	**MW647005**	‒	This work
* R. grisea *	UE2005.08.16-01	Sweden	DQ422030	DQ422030	‒	‒	Buyck et al. 2008
* R. grisea *	FH12234	Germany	KT934006	KT933867	‒	‒	Looney et al. 2016
* R. heterophylla *	UE20.08.2004-2	Sweden	DQ422006	DQ422006	‒	‒	Buyck et al. 2008
* R. ilicis *	563IC52	Europe	AY061682	‒	‒	‒	Miller and Buyck 2002
* R. lakhanpalii *	AG 17-1584	India	MN262088	‒	‒	‒	Ghosh et al. 2020
* R. lakhanpalii *	RITF2600	China	**MW646983**	**MW646992**	**MW647006**	**MW650850**	This work
* R. lotus *	RITF499	China	MK860699	MW397129	MK860706	‒	Song et al. 2019
*** R. luofuensis ***	**RITF4706**	**China**	**MW646973**	**MW646985**	**MW646996**	**MW650842**	**This work**
*** R. luofuensis ***	**RITF4707**	**China**	**MW646974**	**MW646986**	**MW646997**	**MW650843**	**This work**
*** R. luofuensis ***	**RITF4708**	**China**	**MW646975**	**MW646987**	**MW646998**	**MW650844**	**This work**
*** R. luofuensis ***	**RITF4712**	**China**	**MW646976**	**MW646988**	**MW646999**	**MW650845**	**This work**
*** R. luofuensis ***	**RITF4714**	**China**	**MW646977**	**MW646989**	**MW647000**	**MW650846**	**This work**
*R.maguanensi*s	XHW4765	China	MH724918	MH714537	‒	MH939983	Wang et al. 2019
* R. mustelina *	FH12226	Germany	KT934005	KT933866	‒	‒	Looney et al. 2016
* R. orientipurpurea *	SFC20170819-08	South Korea	MT017550	‒	MT196926	MT199651	Wisitrassameewong et al. 2020
* R. orientipurpurea *	SFC20170725-37	South Korea	MT017548	‒	MT196927	MT199652	Wisitrassameewong et al. 2020
* R. pallidula *	RITF2613	China	MH027958	MH027960	MW403845	**MW650852**	Chen et al. 2019, This work
* R. pallidula *	RITF3331	China	MH027959	MH027961	MW403846	**MW650853**	Chen et al. 2020, This work
* R. parvovirescens *	SDRM 6280	USA	MK532789	‒	‒	‒	Unpublished
* R. phloginea *	CNX530524068	China	MK860701	MK860704	MK860708	MK894877	Song et al. 2019
* R. phloginea *	CNX530524304	China	MK860700	MK860703	MK860707	MK894876	Song et al. 2019
* R. prasina *	HMAS 281232	China	MH454351	‒	‒	‒	Hyde et al. 2019
* R. pseudobubalina *	GDGM70632	China	MF433036	‒	‒	‒	Li et al. 2019
*** R. subbubalina ***	**RITF4710**	**China**	**MW646978**	**MW646990**	**MW647001**	**MW650847**	**This work**
*** R. subbubalina ***	**RITF4715**	**China**	**MW646979**	**MW646991**	**MW647002**	**MW650848**	**This work**
* R. subpallidirosea *	RITF4083	China	MK860697	MK860702	MK860705	MK894875	Song et al. 2019
*R.substriat*a	XHW4766	China	MH724921	MH714540	‒	MH939986	Wang et al. 2019
* R. vesca *	RITF5038	China	**MW646984**	‒	**MW647007**	**MW650851**	**This work**
* R. vesca *	BPL284	USA	KT933978	KT933839	‒	‒	Looney et al. 2016
* R. virescens *	HJB9989	Belgium	DQ422014	DQ422014	‒	‒	Buyck et al. 2008
* R. viridicinnamomea *	K15091418	China	MK049972	‒	‒	MN617850	Yuan et al. 2019
* R. viridicinnamomea *	RITF3324	China	MW397098	MW397130	MW403847	‒	Unpublished
* R. viridirubrolimbata *	HBAU 15011	China	MT337526	‒	‒	‒	Deng et al. 2020
* R. werneri *	IB1997/0786	Europe	DQ422021	DQ422021	‒	‒	Unpublished
* R. xanthovirens *	GDGM 71145	China	MG786056	‒	‒	‒	Song et al. 2018b

### Phylogenetic analysis

Species in the subg. Heterophyllidia with high similarity to our new species and partially representative species that are closely related to subsect. Heterophyllinae (Fr.) Jul. Schäff. and subsect. Virescentinae Singer were selected for phylogenetic analyses. *Russulamaguanensis* J. Wang, X.H. Wang, Buyck & T. Bau and *R.substriata* J. Wang, X.H. Wang, Buyck & T. Bau were used as outgroup. NCBI accession numbers and references of sequences used in the phylogenetic tree are listed in Table 1. Initial sequence alignment was performed using the online version MAFFT 7.0 (http://mafft.cbrc.jp/alignment/server/) with manual evaluations and adjustments in BioEdit when necessary to obtain reliable and reasonable results (Hall 1999). The final aligned result was submitted to TreeBASE (S27792). Maximum likelihood (ML) and Bayesian analysis (BA) were implemented for the phylogenetic analyses. The maximum likelihood was carried out by using RAxML-HPC2 on XSEDE (8.2.12) through the CIPRES Science Gateway (www.phylo.org). The ML analysis was executed by applying the rapid bootstrap algorithm with 1000 replicates to affirm the consistency of the results under the GAMMA model. Bootstrap support (BS) ≥70% on the final tree was regarded as significant. The BA was performed on XSEDE (MrBayes 3.2.7a) through the CIPRES Science Gateway (www.phylo.org) under the GTR model. Four independent Markov chains were run for a total of 50000000 generations, trees were sampled every 100 generations, and the first 25% of the trees were discarded as the burn-in phase of each analysis. The Bayesian posterior probability (PP) values were obtained from the 50% majority-rule consensus trees, and nodes with PP ≥0.95 were considered to be significantly supported.

## Results

### Phylogeny

**Figure 1.**
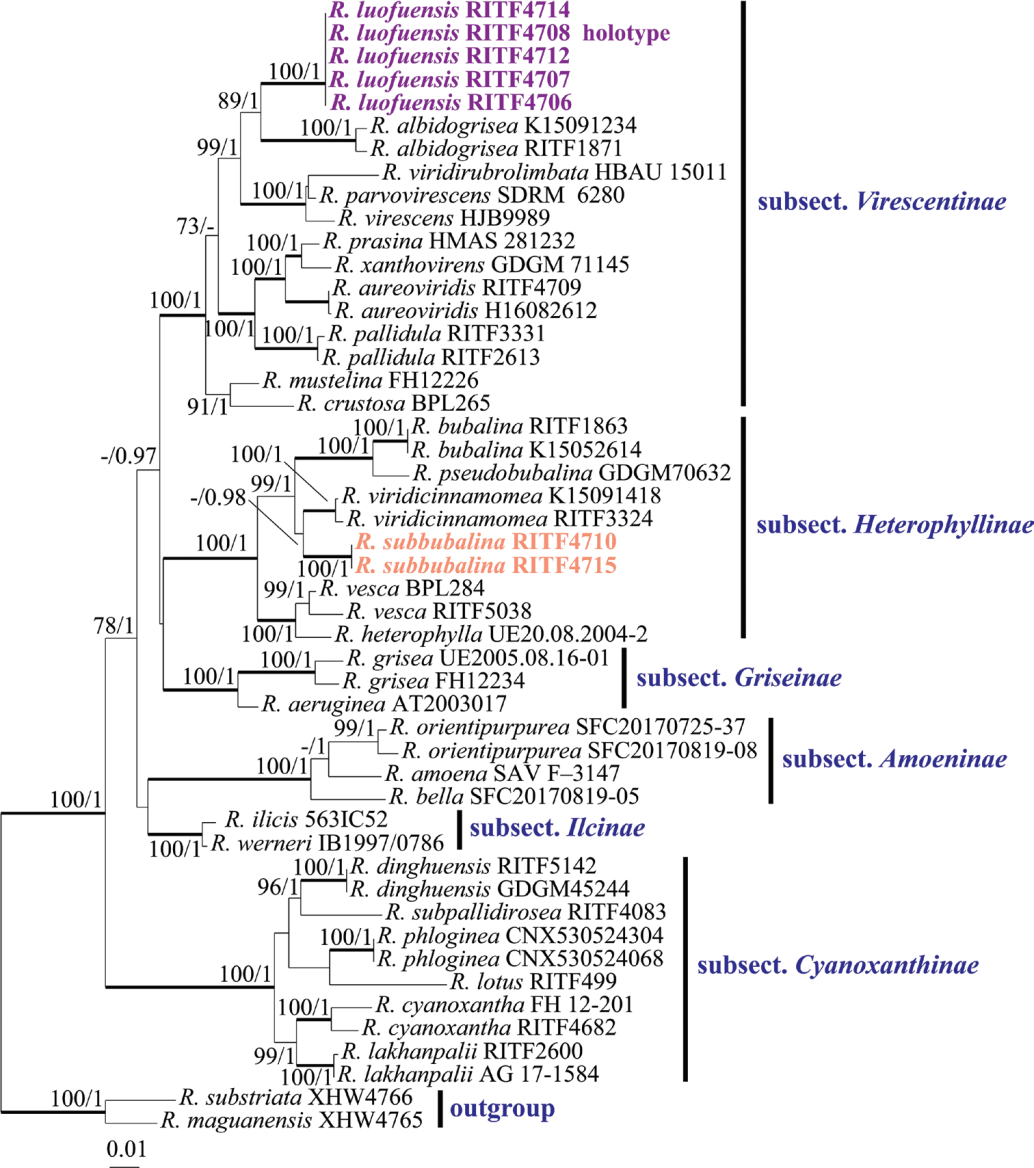
Phylogenetic tree of based on the ITS-nrLS-mtSSU-*TEF1* dataset. Species in the subg. Heterophyllidia with high similarity to our new species and partially representative species that are closely related to subsect. Heterophyllinae and subsect. Virescentinae were selected. *Russulamaguanensis* and *R.substriata* were used as outgroup. Bootstrap support (BS) ≥70% are shown. Bayesian Posterior Probabilities (PP) ≥0.95 are given. Infrageneric classification follows Buyck et al. (2018).

Both the ML analysis and BA of combined ITS-nrLSU-mtSSU-*TEF1* sequences dataset resulted in similar tree topologies, and only the ML tree is shown in Fig. 1. The posterior probabilities for the BA are also shown along the branches. The phylogenetic analyses confirmed that both subsect. Virescentinae and subsect. Heterophyllinae were a monophyletic group; each strongly supported by BS (100%) and PP (1). Additionally, the monophyly of the remaining 4 subsections of subg. Heterophyllidia was also significantly supported.

The samples of the two new species, *R.luofuensis* and *R.subbubalina*, formed each a strongly supported clade (BS 100%, PP 1.00) and were clearly distinct from known and sequenced species of the subg. Heterophyllidia. *R.luofuensis* clustered together with Chinese species *R.albidogrisea* J. W. Li & L. H. Qiu, which is sister to a clade comprising *R.viridirubrolimbata* J. Z. Ying, *R.parvovirescens* Buyck, D. Mitch. & Parrent and *R.virescens* (Schaeff.) Fr. with 99% bootstrap support and 1.00 posterior probabilities. Our second species, *R.subbubalina* clustered with Chinese species *R.viridicinnamomea* F. Yuan & Y. Song and formed a sister clade to two Chinese species (*R.bubalina* J.W. Li & L.H. Qiu and *R.pseudobubalina* J.W. Li & L.H. Qiu) with 99% bootstrap support and 1.00 posterior probabilities.

## Taxonomy

### Russula luofuensis

Taxon classificationFungiRussulalesRussulaceae

B. Chen & J. F. Liang sp. nov.

27D67B5D-5FAB-541A-ACD9-140BCA3DFB80

MB838836

Figs 2A–D , 3 and 4

#### Diagnosis.

Basidiomata medium-sized to large; grayish yellow to brownish orange pileus center, purplish gray to grayish magenta towards the margin, surface cracking and broken into small golden-brown patches, peeling to 1/2 of the radius; subglobose to broadly ellipsoid basidiospores with warts fused in short or long chains; hymenial gloeocystidia mainly clavate; suprapellis composed of hyphal extremities with inflated, ellipsoid or globose cells and attenuated terminal cell; pileocystidia always one-celled, apically typically obtuse.

#### Holotype.

China. Guangdong Province, Huizhou City, Boluo County, Luofu Mountain Provincial Nature Reserve, 23°15'47.13"N, 114°3'45.42"E, 90 m asl., in mixed Fagaceae forests of *Cyclobalanopsis* and *Castanopsis*, 22 August 2020, leg. CB446 (RITF4708).

#### Etymology.

The species name refers to the type locality, Luofu Mountain Provincial Nature Reserve.

#### Description.

**Basidiomata** medium-sized to large; pileus 35–80 mm in diameter; initially hemispheric when young, applanate to convex, convex with a depressed center after mature; margin incurved, not cracked, striation short and inconspicuous; surface dry, glabrous, peeling to 1/2 of the radius, cracking and broken into small golden-brown patches, patches crowded towards the center, with smaller patches towards the margin; grayish yellow (4B5) to brownish orange (5C5) in the center, purplish gray (13B2) to grayish magenta (13B3) towards the margin. **Lamellae** adnate to subfree, 2–4 mm deep, 8–10 at 1 cm near the pileus margin, white (1A1) to cream; lamellulae absent; furcations occasional near the stipe; edge entire and concolor. **Stipe** 30–50 × 10–25 mm, cylindrical, slightly inflated towards the base, white (1A1), with yellowish (2A2) tinge at the base, and medulla initially stuffed becoming hollow. **Context** 2–3 mm thick in half of the pileus radius, white (1A1), unchanging when bruised, taste mild, odor inconspicuous. **Spore print** pale yellowish (2A2).

**Figure 2.**
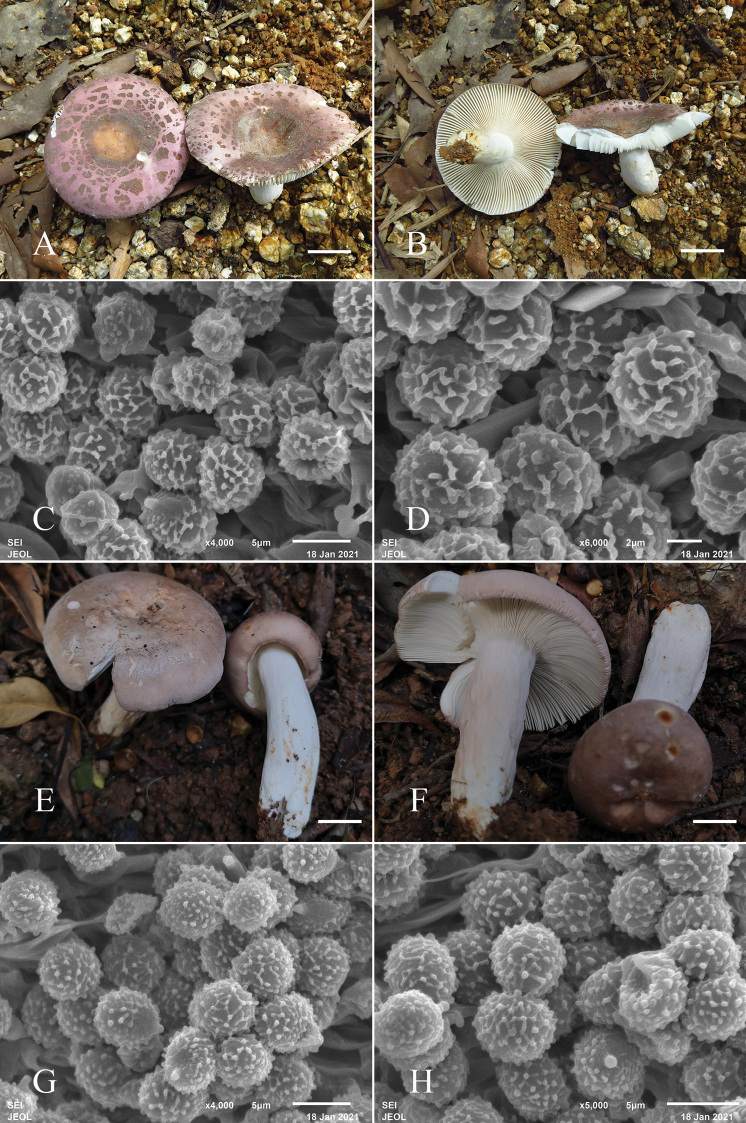
Fruiting bodies (**A, B**) and basidiospores (**C, D**) of *Russulaluofuensis* (RITF4708). Fruiting bodies (**E, F**) and basidiospores (**G, H**) of *R.subbubalina* (RITF 3715). Scale bars: 20 mm (**A, B, E, F)**.

**Basidiospores** (5.0–)5.8–6.6–7.5(–8.6) × (4.5–)5.4–6.2–7.0(–8.0) μm, Q = (1.0–)1.02–1.08–1.14(–1.26), subglobose to broadly ellipsoid; ornamentation of medium-sized, moderately distant to dense [6–8(–9) in a 3 μm diameter circle] amyloid warts or spines, 0.3–0.6 μm high, locally reticulate, frequently fused in short or long chains [2–3(–4) in the circle], occasionally to frequently connected by line connections [1–2(–3) in the circle]; suprahilar spot medium-sized, amyloid. **Basidia** (35.0–)36.7–39.8–42.8(–45.5) × (9.0–)9.5–10.0–10.5(–11.2) μm, mostly 4-spored, some 2- and 3-spored, clavate; basidiola clavate or subcylindrical, ca. 6.5–11.5 μm wide. **Hymenial gloeocystidia on lamellae sides** dispersed to moderately numerous, ca. 600–900/mm^2^, (59.0)63.2–71.3–79.3(83.6) × (7.0)7.7–8.8–9.9(10.5) μm, clavate or narrowly clavate, apically mainly obtuse, occasionally acute, often with 3–10 μm long appendage, thin-walled; contents heteromorphous or granulose, mainly in the middle and upper part, turning reddish black in SV. **Hymenial gloeocystidia on lamellae edges** often smaller, (49.5–)56.2–64.3–72.4(–80.2) × (6.2–)7.3–8.3–9.4(–10.0) μm, clavate, or subcylindrical, sometimes fusiform, apically mainly obtuse, occasionally mucronate, sometimes with 3–6 μm long appendage thin-walled; contents heteromorphous, turning reddish black in SV. **Marginal cells** (15.2–)19.8–23.5–27.2(–30.6) × (3.5–)4.0–4.8–5.6(–7.0) μm, subcylindrical or clavate, often flexuous. **Pileipellis** orthochromatic in cresyl blue, not sharply delimited from the underlying context, 260–300 μm deep, two-layered; suprapellis 120–150 μm deep, hyphal endings composed of inflated, ellipsoid or globose cells with attenuated terminal cells; subpellis 120–160 μm deep, composed of repent, intricate, 2–6 μm wide hyphae. Hyphal terminations near the pileus margin typically unbranched, occasionally flexuous, thin-walled; terminal cells (9.2–)18.6–28.2–37.8(–50.8) × (3.2–)3.9–5.0–6.1(–8.2) μm, mainly narrowly lageniform, occasionally clavate or cylindrical, apically attenuated or constricted, occasionally obtuse; subterminal cells frequently shorter and wider, ca. 4–9 μm wide, typically unbranched. Hyphal terminations near the pileus center similar to those near the pileus margin; terminal cells (10.2–)18.4–27.4–36.4(–44.8) × (3.2–)3.6–4.7–5.8(–6.8) μm, mainly lageniform, occasionally fusiform or subcylindrical, apically attenuated or constricted; subterminal cells often shorter and wider, rarely branched, ca. 4–7 μm wide. **Pileocystidia** near the pileus margin always one-celled, (23.3–)27.9–35.0–42.2(–47.5) ×3.5–4.8–6.0(–8.3) μm, mainly clavate, occasionally subcylindrical or fusiform, apically typically obtuse, occasionally acute, often with round or ellipsoid, 3–6 μm long appendage, thin-walled; contents heteromorphous or granulose, turning reddish black in SV. Pileocystidia near the pileus center similar in size, always one-celled, (24.6–)27.2–34.8–42.5(–48.2) × 3.0–4.2–5.4(–6.8) μm, thin-walled, mainly clavate, occasionally fusiform, apically often obtuse or occasionally acute, occasionally with 2–4 μm long appendage, contents heteromorphous or granulose, turning reddish black in SV. **Cystidioid hyphae** In subpellis and context with heteromorphous contents, oleiferous hyphae in subpellis with refringent contents.

**Figure 3.**
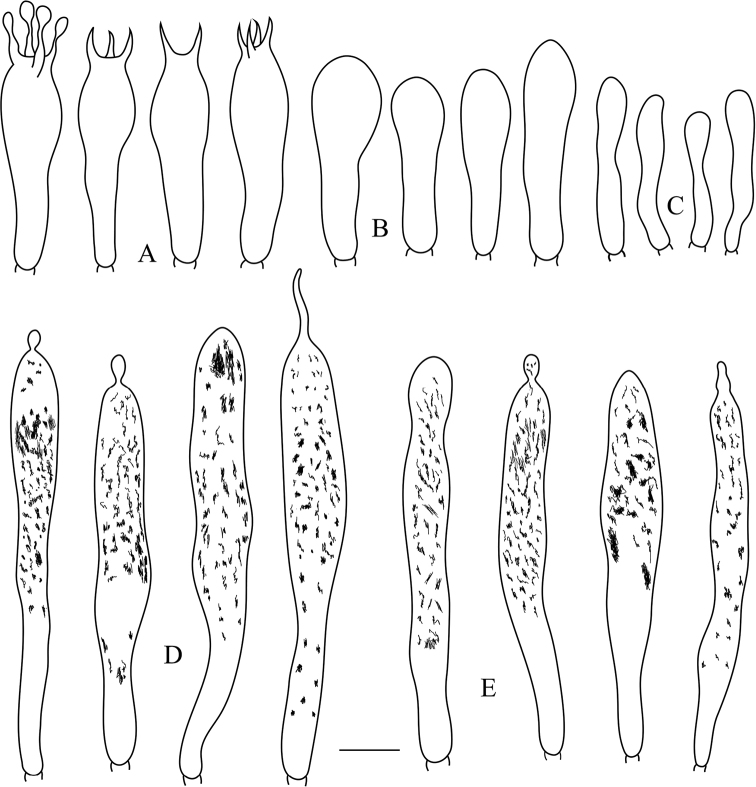
*Russulaluofuensis* (RITF 4708) **A** basidia **B** basidiola **C** marginal cells **D** hymenial gloeocystidia on lamellae sides **E** hymenial gloeocystidia on lamellae edges. Scale bar: 10 μm.

#### Additional specimens examined.

China. Guangdong Province, Huizhou City, Boluo County, Luofu Mountain Provincial Nature Reserve, 23°15'44.11"N, 114°3'16.77"E, 120 m asl., in mixed Fagaceae forests of *Cyclobalanopsis* and *Castanopsis*, 22 August 2020, leg. CB444 (RITF4706); ibid., 22 August 2020, leg. CB445 (RITF4707); ibid., 22 August 2020, leg. CB450 (RITF4712); ibid., 22 August 2020, leg. CB452 (RITF4714).

#### Notes.

The combination of morphological features and phylogenetic analysis place *R.luofuensis* in subsect. Virescentinae. Phylogenetically, our new species *R.luofuensis* is clustered with *R.albidogrisea* with 89% bootstrap support and 1.00 posterior probabilities, which is also from Guangdong Province of China. However, *R.albidogrisea* differs from *R.luofuensis* in having a white to grayish pileus with acute, even to slightly undulate margin, often smaller basidiospores [(5.1–)5.3–5.6–6.0(–6.4) × (4.6–)4.8–5.1–5.3(–5.6) μm], longer hymenial gloeocystidia on lamellae sides (35–50 × 5–11 μm) and hymenial gloeocystidia on lamellae edges (37–46 × 9–12 μm, Das et al. 2017).

Given cracking surface, *R.viridirubrolimbata*, *R.parvovirescens*, *R.virescens* and *R.crustosa* Peck of subsect. Virescentinae resemble *R.luofuensis*. However, *R.viridirubrolimbata*, originally described from China, can be distinguished by a light yellowish olive to yellowish olive pileus center with a pinkish red to light jasper red margin and absence of hymenial gloeocystidia on lamellae edges (Ying 1983; Deng et al. 2020). The American species *R.parvovirescens* possesses a greenish brown to metallic bluish green pileus with green patches (Buyck et al. 2006). *Russulavirescens* (originally reported from Europe) is distinct in its green to yellowish green pileus (Sarnari 1998). *Russulaalbidogrisea*, originally reported from North America, has a brownish-yellow, greenish or subolivaceous pileus with small spot-like areolae or pseudo-verrucae, shorter basidia [(29–)30–32–33.5(–35) × (7.5–)8–9.5–10.5(–11) μm] and absence of hymenial gloeocystidia on the lamellar edges (Adamčík et al. 2018).

**Figure 4.**
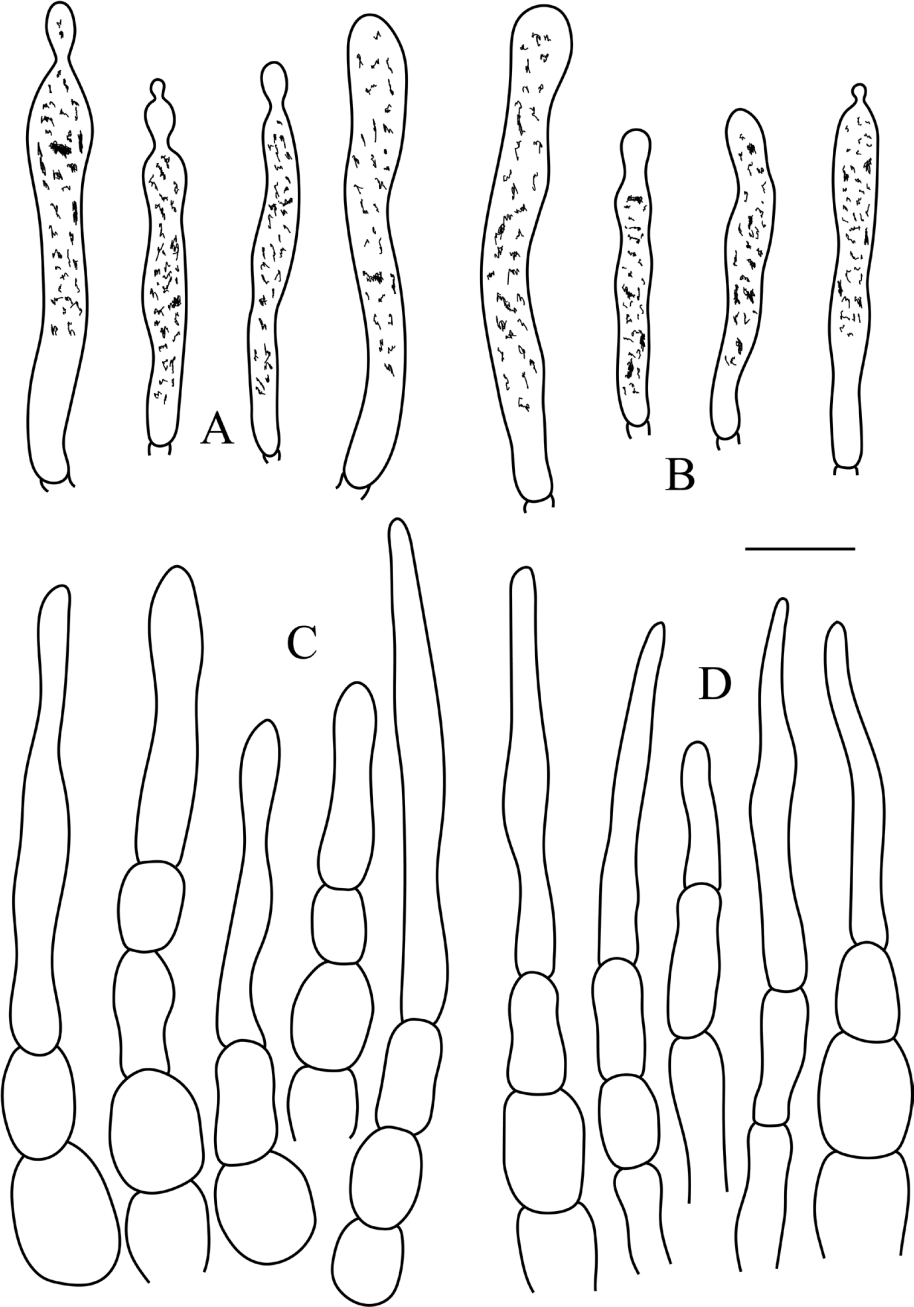
*Russulaluofuensis* (RITF 4708) **A** pileocystidia near the pileus margin **B** pileocystidia near the pileus center **C** hyphal terminations near the pileus margin **D** hyphal terminations near the pileus center. Scale bar: 10 μm.

### Russula subbubalina

Taxon classificationFungiRussulalesRussulaceae

B. Chen & J. F. Liang sp. nov.

91CC489C-BA99-53AF-91C1-B4F7B6F73FF9

MB838837

Figs 2E–H , 5 and 6

#### Diagnosis.

Basidiomata medium-sized to large; dark salmon pileus with rusty spots when young, blanched almond with a cracked margin after maturation, surface pruinose in some parts; adnate to slightly adnexed lamellae; subglobose to broadly ellipsoid basidiospores with warts fused in short or long chains and frequently connected by line connections; clavate or ellipsoid basidiola; hymenial gloeocystidia clavate or fusiform, apically mainly obtuse; suprapellis with hyphal ends composed of inflated or ellipsoid cells and attenuated terminal cell; pileocystidia mainly clavate, apically typically obtuse, sometimes with round or ellipsoid appendage.

#### Holotype.

China. Guangdong Province, Huizhou City, Boluo County, Luofu Mountain Provincial Nature Reserve, 23°15'43.80"N, 114°3'5.40"E, 220 m asl., in mixed Fagaceae forests of *Cyclobalanopsis* and *Castanopsis*, 22 August 2020, leg. CB448 (RITF4710).

#### Etymology.

Referred to its morphological resemblance to *R.bubalina*.

#### Description.

**Basidiomata** medium-sized to large; pileus 50–100 mm in diameter; initially hemispheric when young, applanate to convex, convex with a slightly depressed center after mature; margin incurved, cracked with age, striation short and inconspicuous; surface dry, glabrous, peeling to 1/4 of the radius, pruinose in some part; dark salmon with rusty spots when young, blanched almond after maturation, shallower at the margin. **Lamellae** adnate to slightly adnexed, 3–5 mm deep, 11–13 at 1 cm near the pileus margin, white (1A1) to cream; lamellulae sometimes present and irregular in length; furcations present especially near the stipe; edge entire and concolor. **Stipe** 30–55 × 5–15 mm, cylindrical, slightly inflated towards the base, white (1A1) to blanched almond, with rusty tinge towards the base, and medulla initially stuffed becoming hollow. **Context** 3–4 mm thick in half of the pileus radius, white (1A1), unchanging when bruised, taste mild, odor inconspicuous. **Spore print** white (1A1) to cream.

**Figure 5.**
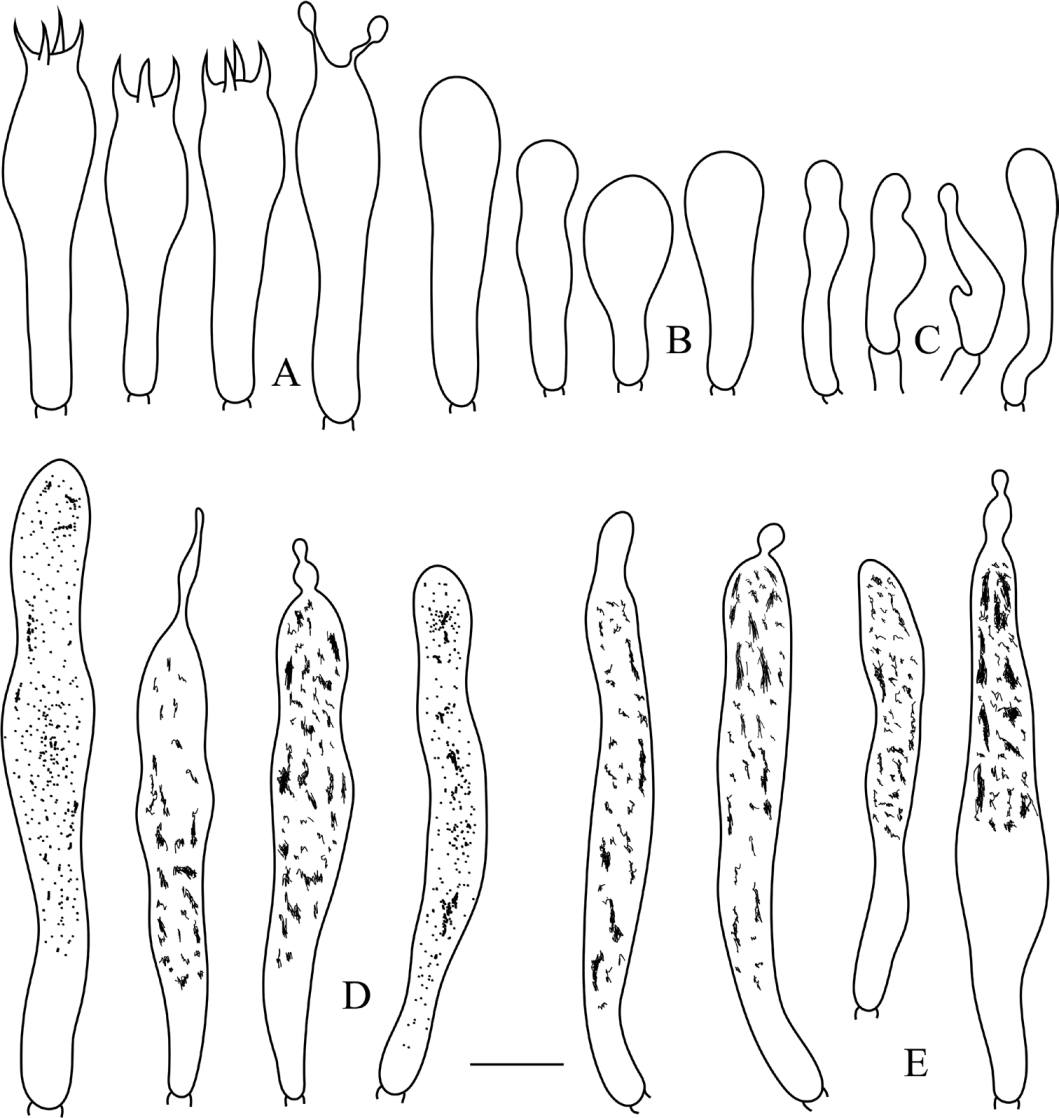
*Russulasubbubalina* (RITF 4710) **A** basidia **B** basidiola **C** marginal cells **D** hymenial gloeocystidia on lamellae sides **E** hymenial gloeocystidia on lamellae edges. Scale bar: 10 μm.

**Basidiospores** (5.2–)5.6–6.2–6.8(–7.2) × (4.5–)4.9–5.3–5.7(–6.2) μm, Q = (1.0–)1.08–1.17–1.25(–1.38), subglobose to broadly ellipsoid; ornamentation of relatively small, moderately distant to dense [6–8(–9) in a 3 μm diameter circle] amyloid warts or spines, 0.3–0.5 μm high, locally reticulate, fused in short or long chains [2–3(–4) in the circle], frequently connected by line connections [3–4(–5) in the circle]; suprahilar spot medium-sized, amyloid. **Basidia** (30.5–)31.7–34.8–37.8(–43.0) × (6.3–)7.5–8.1–8.8(–9.4) μm, mostly 4-spored, some 2- and 3-spored, clavate; basidiola clavate or ellipsoid, ca. 5.5–10 μm wide. **Hymenial gloeocystidia on lamellae sides** Moderately numerous, ca. 800–1000/mm^2^, (41.0)49.1–56.7–64.3(68.5) × (6.5)7.2–8.1–9.0(10.0) μm, clavate or fusiform, apically mainly obtuse, occasionally acute, sometimes with 4–10 μm long appendage, thin-walled; contents heteromorphous or granulose, turning reddish black in SV. **Hymenial gloeocystidia on lamellae edges** Often longer, (40.5–)52.6–63.0–73.5(–83.6) × (4.6–)6.7–8.1–9.6(–10.8) μm, mainly clavate, occasionally fusiform, apically typically obtuse, sometimes with 3–8 μm long appendage, thin-walled; contents heteromorphous-crystalline, turning reddish black in SV. **Marginal cells** (14.0–)19.0–23.4–27.7(–34.2) × (3.4–)3.7–4.5–5.3(–5.8) μm, clavate, lageniform or fusiform, often flexuous. **Pileipellis** Orthochromatic in cresyl blue, sharply delimited from the underlying context, 400–450 μm deep, two-layered; suprapellis180–200 μm deep, hyphal endings composed of inflated or ellipsoid cells with attenuated terminal cells; subpellis 240–260 μm deep, composed of horizontally oriented, relatively dense, intricate, 3–6 μm wide hyphae. Hyphal terminations near the pileus margin sometimes branched, occasionally flexuous, thin-walled; terminal cells (14.8)20.9–26.6–32.3(38.0) × 3.5–4.0–4.6(5.5) μm, mainly narrowly lageniform, occasionally cylindrical, apically attenuated or constricted; subterminal cells frequently shorter and wider ca. 3–8 μm wide, occasionally branched. Hyphal terminations near the pileus center similar to those near the pileus margin; terminal cells (14.3–)17.5–22.7–27.8(–33.7) × (3.4–)3.7–4.1–4.6(–5.0) μm, lageniform, clavate or cylindrical, apically attenuated or constricted, sometimes obtuse; subterminal cells often wider, rarely branched, ca. 4–8 μm wide. **Pileocystidia** near the pileus margin always one-celled, (27.9–)35.1–40.5–45.9(–48.9) × (3.8–)4.2–4.7–5.3(–5.7) μm, mainly clavate, occasionally fusiform, apically typically obtuse, sometimes with round or ellipsoid 2–6 μm long appendage, thin-walled; contents heteromorphous, turning reddish black in SV. Pileocystidia near the pileus center similar in shape, always one-celled, (23.7–)25.6–31.8–38.0(–46.0) × (3.3–)4.2–4.8–5.4(–6.0) μm, thin-walled, mainly clavate, occasionally fusiform or subcylindrical, apically typically obtuse, sometimes with 4–6 μm long appendage, contents granulose, turning reddish in SV. **Cystidioid hyphae** In subpellis and context with granulose contents, oleiferous hyphae frequent in subpellis with yellowish contents.

**Figure 6.**
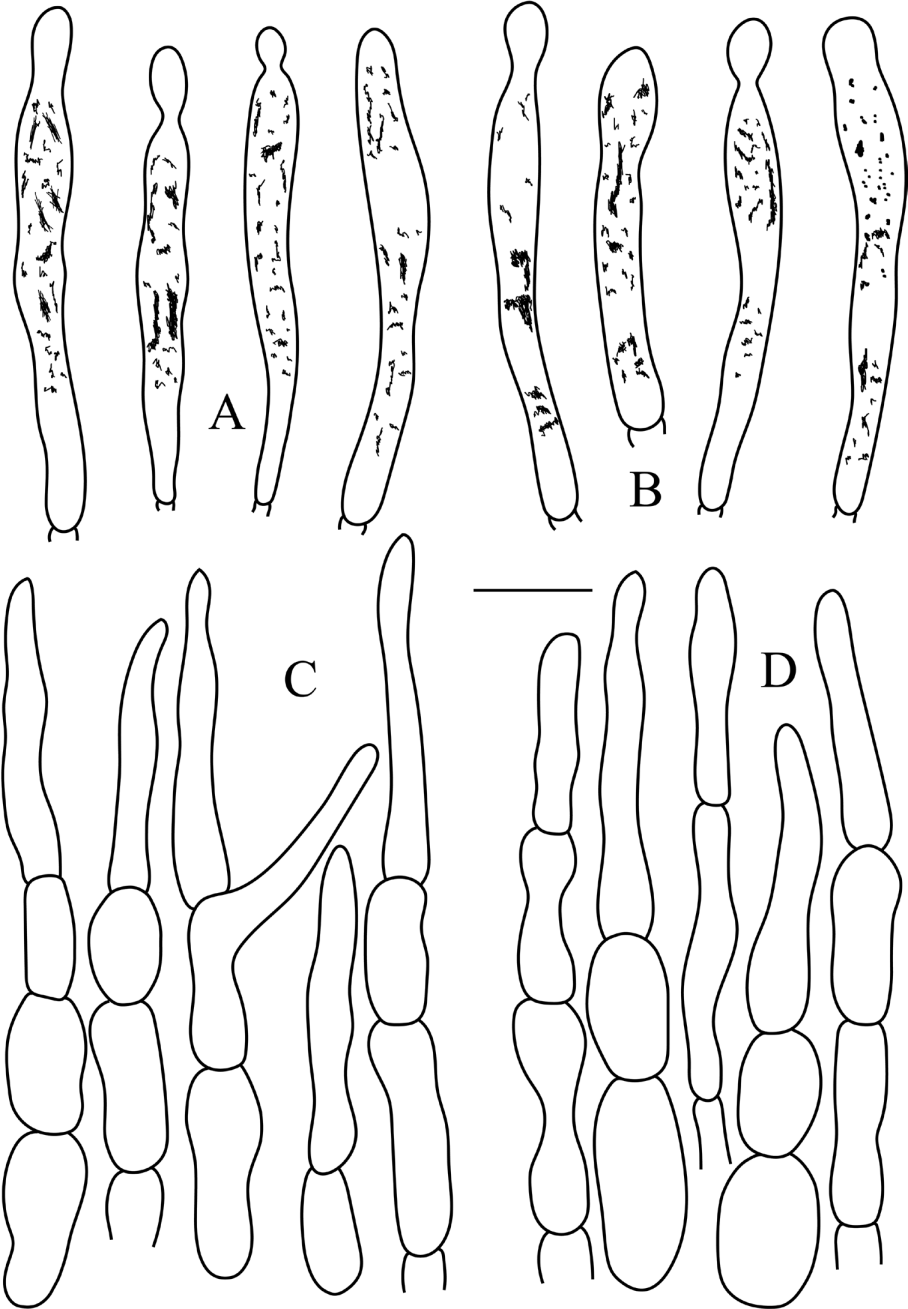
*Russulasubbubalina* (RITF 4710) **A** pileocystidia near the pileus margin **B** pileocystidia near the pileus center **C** hyphal terminations near the pileus margin **D** hyphal terminations near the pileus center. Scale bar: 10 μm.

#### Additional specimens examined.

China. Guangdong Province, Huizhou City, Boluo County, Luofu Mountain Provincial Nature Reserve, 23°15'41.70"N, 114°3'5.21"E, 240 m asl., in mixed Fagaceae forests of *Cyclobalanopsis* and *Castanopsis*, 22 August 2020, leg. CB453 (RITF4710).

#### Notes.

Both morphology and phylogeny place *R.subbubalina* clearly in subsect. Heterophyllinae. In our phylogenetic tree, *R.viridicinnamomea* is the sister taxon to *R.subbubalina* but differs from it by the typically smaller basidiomata (30–50 μm), an emerald green-tinged buff pileus with undulate and tearing margin and longer hymenial gloeocystidia on the lamellae edges (36.5–63 × 4–12 μm, Yuan et al. 2019).

Morphologically, *R.subbubalina* may be confused in the field with two recently reported new species: *R.bubalina* and *R.pseudobubalina* also from Guangdong Province of China. However, *R.bubalina* has the typically smaller basidiomata (35–54 μm), a striate pileus margin and basidiospores with warty ornamentations not forming reticulum (Li et al. 2019), whereas *R.pseudobubalina* possesses the typically smaller basidiomata (31–46 μm), never forked lamellae, basidiospores with isolated warts, and often shorter hymenial gloeocystidia on the lamellae edges (23.4–37.8–65.5 × 6.2–8.3–10.0 μm, Li et al. 2019).

## Supplementary Material

XML Treatment for Russula luofuensis

XML Treatment for Russula subbubalina
